# Quantitative analysis of choroidal vasculature in polypoidal choroidal vasculopathy using ultra-widefield indocyanine green angiography

**DOI:** 10.1038/s41598-020-75506-7

**Published:** 2020-10-26

**Authors:** Gahyung Ryu, Cheolwon Moon, Jano van Hemert, Min Sagong

**Affiliations:** 1 Department of Ophthalmology, Yeungnam University College of Medicine, #170 Hyunchungro, Nam-gu, Daegu, 42415 South Korea; 2 Yeungnam Eye Center, Yeungnam University Hospital, Daegu, South Korea; 3 Optos PLC, Dunfermline, UK

**Keywords:** Eye diseases, Macular degeneration, Translational research, Medical research, Pathogenesis, Prognostic markers, Medical imaging, Blood flow

## Abstract

Polypoidal choroidal vasculopathy (PCV) is a common choroidal vascular disease particularly in Asians. However, the underlying pathogenesis of PCV is still yet to be fully elucidated, and the correlation between choroidal vasculature and treatment response of PCV are poorly understood. Accordingly, we sought to find clues to understand the pathogenesis and prognosis of PCV by quantitatively evaluating choroidal vasculature from the entire fundus using ultra-widefield (UWF) indocyanine green angiography (ICGA). In this study, 32 eyes from 29 patients with treatment naïve PCV and 30 eyes from 30 healthy control participants were enrolled. Choroidal vascular density (CVD) of PCV eyes was higher than normal eyes in majority regions including the periphery. CVD was positively correlated with choroidal thickness and choroidal hyperpermeability, supporting that the pathogenesis of PCV may include choroidal congestion and dilatation. Thicker choroid and higher CVD were also correlated with poor treatment response after anti-VEGF injections. The CVD, quantified from UWF ICGA can also be used as an effective image biomarker to predict the treatment response in PCV.

## Introduction

Polypoidal choroidal vasculopathy (PCV) is believed to be a subtype of neovascular age-related macular degeneration^1–4^. Currently, it has been proposed to be described as aneurysmal type 1 neovascularization, which is associated with an abnormal branching vascular network (BVN) with aneurysmal dilatations referred to as polyps^5,6^. Clinically, it is characterized by nodular, orange-red vascular lesions and recurrent serosanguineous detachments of the retinal pigment epithelium (RPE) and neurosensory retina^7^.

Recent studies have suggested that pathogenesis of PCV may include a pachychoroid-driven process involving choroidal congestion, manifested by choroidal thickening and hyperpermeability^8–13^. Although the choroidal thickness is generally increased in eyes with PCV, a wide variation in choroidal thickness has been reported^14–16^. And also, thick choroid and presence of choroidal vascular hyperpermeability are known to be poor prognostic factors after anti-VEGF therapy for PCV^9,17–21^. Thus, evaluation of the choroid in PCV has important value in predicting the prognosis of treatment response and supporting disease pathogenesis in addition to simply presenting the clinical and imaging features of the disease.

However, the findings of previous studies were mostly based on cross-sectional images of enhanced depth imaging (EDI) or en face optical coherence tomography (OCT), which demonstrate only limited field-of-view. Chung et al. demonstrated the association between engorgement of the vortex vein and the development of PCV using a montage of images taken by conventional indocyanine green angiography (ICGA) with a confocal laser scanning system^22^. Although the study was the first to report the choroidal vascular features of PCV from the extended area, quantitative analysis of choroidal vasculature was not available since the montage image does not permit simultaneous imaging of the posterior pole and periphery.

Ultra-wide field (UWF) retinal imaging devices provide images to the far periphery, to be captured in a single image. Since then, several studies have attempted quantitative analysis of choroidal vasculature in the patients with PCV, but these was a potential limitation due to warping inherent in UWF imaging^23,24^. With advances in UWF software, the real physical area can be calculated by accounting for non-linear distortion, defined by its location in the image. Thus, we sought to evaluate the precise area and density of choroidal vasculature using UWF ICGA over the whole fundus and in each prespecified region in the PCV patients. In addition, we evaluated the relationship between the choroidal vascular density (CVD) with baseline characteristics, including clinical and OCT features, and investigated which baseline parameters were associated with the response to anti-vascular endothelial growth factor (VEGF) treatment.

## Results

Thirty-two eyes from 29 patients with PCV and 30 eyes from 30 age-matched normal subjects were enrolled for this study. There were no significant differences in age, sex, and refractory error between the two groups (Table 1). Mean best-corrected visual acuity (BCVA) was 0.51 ± 0.34 logMAR in the PCV group and 0.06 ± 0.08 logMAR in the control group (*p* < 0.001) (Table 1). In the PCV group, the mean number of polyps was 1.5 ± 0.7, and the mean greatest linear dimension was 2549.4 ± 1086.6 µm. The incidence of choroidal vascular hyperpermeability was 34.4%.

**Table 1:** Baseline demographics of the PCV and control groups.

	PCV (n = 32)	Control (n = 30)	*P* value
Age, years	68.5 ± 9.8	64.8 ± 5.0	0.114^a^
Sex, n (%)			0.567^b^
Male	22 (68.8%)	21 (70.0%)	
Female	10 (31.3%)	9 (30.0%)	
Refractive error, diopter	0.24 ± 1.69	0.59 ± 0.96	0.549^a^
Baseline BCVA, logMAR	0.51 ± 0.34	0.06 ± 0.08	< 0.001^a^
Baseline CMT, µm	417.97 ± 130.15	273.00 ± 45.59	< 0.001^a^
SFCT, µm	316.72 ± 94.49	232.05 ± 63.43	0.001^a^
Haller’s layer	242.50 ± 91.76	141.05 ± 36.51	< 0.001^a^
Choriocapillaris-Sattler's layer	74.22 ± 22.68	91.00 ± 38.72	0.181^a^
Choroidal vascular area, mm^2^	140.20 ± 19.25	132.44 ± 17.56	0.109^a^
Macular region (< 3 mm)	7.30 ± 0.60	6.85 ± 0.71	0.030^a^
Near-peripheral region (3 ~ 10 mm)	67.21 ± 6.83	60.80 ± 7.68	0.003^a^
Mid-peripheral region (10 ~ 15 mm)	54.04 ± 14.75	52.63 ± 12.82	0.549^a^
Far-peripheral region (> 15 mm)	11.65 ± 9.72	12.17 ± 9.83	0.881^a^
Choroidal vascular density, %	27.15 ± 1.86	25.17 ± 2.05	0.002^a^
Macular region (< 3 mm)	25.80 ± 2.12	24.22 ± 2.50	0.029^a^
Near-peripheral region (3 ~ 10 mm)	25.72 ± 1.98	23.43 ± 1.98	< 0.001^a^
Mid-peripheral region (10 ~ 15 mm)	28.67 ± 2.40	26.95 ± 2.49	0.024^a^
Far-peripheral region (> 15 mm)	29.39 ± 2.26	26.94 ± 3.51	0.010^a^

^a^Mann-Whitney test, ^b^Pearson’s chi square test.

BCVA, best-corrected visual acuity; CMT, central macular thickness; PCV, polypoidal choroidal vasculopathy; SFCT, subfoveal choroidal thickness.

### Comparison of tomographic features between PCV and control eyes

Mean central macular thickness (CMT) was 417.97 ± 130.15 µm in the PCV group and 273 ± 45.59 µm in the control group (*p* < 0.001) (Table 1). The subfoveal choroidal thickness (SFCT) was greater in the PCV group than the control group (316.72 ± 94.49 µm vs. 232.05 ± 63.43 µm, *p* = 0.001) (Table 1). In the analysis by layer, the thickness of Haller’s layer was significantly greater in the PCV group (242.50 ± 91.76 µm vs. 141.05 ± 36.51 µm, *p* < 0.001). In comparison, the thickness of the choriocapillaris-Sattler’s layer was thinner in the PCV group than in the normal subjects without statistical significance (74.22 ± 22.68 µm vs. 91.00 ± 38.72 µm, *p* = 0.181) (Table 1).

### Comparison of choroidal vascular features between the PCV and control eyes

The mean of Choroidal vascular area (CVA) in eyes with PCV was 140.20 ± 19.25 mm^2^ for the entire area, 7.30 ± 0.60 mm^2^ for the macular regions (MR), 67.21 ± 6.83 mm^2^ for the near-peripheral region (NPR), 54.04 ± 14.75 mm^2^ for the mid-peripheral region (MPR), and 11.65 ± 9.72 mm^2^ for the far peripheral region (FPR). Although the mean of CVA for the entire area and each region were higher in the PCV group than in the control group, only the CVA in the MR (*p* = 0.030) and NPR (*p* = 0.003) showed statistically significant differences (Table 1).

The mean CVD in eyes with PCV was 27.15 ± 1.86% for the entire area, 25.80 ± 2.12% for the MR, 25.72 ± 1.98% for the NPR, 28.67 ± 2.40% for the MPR, and 29.39 ± 2.26% for the FPR. Compared to the normal group, the mean CVD in the PCV group were significantly higher for the entire area (*p* = 0.002) and each region (*p* = 0.029 for MR; *p* < 0.001 for NPR; *p* = 0.024 for MPR; *p* = 0.010 for FPR) (Table 1).

### Choroidal features of unaffected eyes in unilateral PCV

A total of 22 eyes out of 29 PCV patients were available to be defined as unaffected fellow eyes and to be analyzed. The CVD of the fellow eyes (26.95 ± 1.98% for total; 26.08 ± 2.60 for MR; 25.10 ± 2.10 for NPR; 28.67 ± 2.09 for MPR; 29.14 ± 2.34 for FPR) were significantly higher than control eyes (*p* = 0.021 for total; *p* = 0.019 for MP; *p* = 0.033 for NPR; *p* = 0.027 for MPR; *p* = 0.044 for FPR), but did not differ significantly from the PCV eyes (*p* = 0.638 for total; *p* = 0.884 for MPR; *p* = 0.223 for NPR; *p* = 0.615 for MPR; *p* = 0.115 for FPR). However, the SFCT of the fellow eyes (287.05 ± 67.74 for total SFCT; 194.55 ± 65.96 for Haller thickness) were significantly thicker than the control eyes (*p* = 0.022 for total thickness; *p* = 0.006 for Haller thickness) and significantly thinner than the PCV eyes (*p* = 0.009 for total thickness; *p* = 0.002 for Haller thickness).

### Correlation between CVD and baseline characteristics in PCV

Linear correlation analysis did not find any association between the CVD with age, sex, refractory error, and baseline BCVA. However, baseline CMT, choroidal thickness, and choroidal vascular hyperpermeability were positively associated with CVD.

Baseline CMT showed statistically significant correlation with the total CVD (R = 0.442, *p* < 0.001 for total), and even when analyzed by prespecified regions, it also showed a significant correlation with the CVD in each region (R = 0.287, *p* = 0.028 for MR; R = 0.437, *p* = 0.001 for NPR, R = 0.437, *p* = 0.001 for MPR; R = 0.316, *p* = 0.015 for FPR) (Table 2).

**Table 2:** Choroidal vascular density and associated factors.

	Total		MR (< 3 mm)		NPR (3 ~ 10 mm)		MPR (10 ~ 15 mm)		FPR (> 15 mm)	
	R	*P*^a^	R	*P*^a^	R	*P*^a^	R	*P*^a^	R	*P*^a^
Age	− 0.171	0.183	0.044	0.735	− 0.116	0.369	− 0.225	0.079	− 0.178	0.167
Sex	− 0.053	0.684	− 0.088	0.495	− 0.075	0.562	− 0.003	0.981	− 0.068	0.598
Refractory error	− 0.171	0.183	0.044	0.735	− 0.116	0.369	− 0.225	0.079	− 0.178	0.167
Baseline BCVA	0.131	0.310	− 0.003	0.984	0.199	0.122	0.152	0.240	− 0.048	0.711
Baseline CMT	0.442	< 0.001	0.287	0.028	0.437	0.001	0.437	0.001	0.316	0.015
SFCT	0.448	< 0.001	0.229	0.074	0.449	< 0.001	0.477	< 0.001	0.305	0.016
Haller’s layer	0.504	< 0.001	0.281	0.027	0.510	< 0.001	0.520	< 0.001	0.342	0.007
Choriocapillaris-Sattler's layer	− 0.249	0.051	− 0.249	0.051	− 0.279	0.028	− 0.186	0.147	− 0.165	0.201
Choroidal hyperpermeability	0.481	0.005	0.304	0.091	0.393	0.026	0.473	0.006	0.461	0.008

^a^Pearson correlation.

BCVA, best-corrected visual acuity; CMT, central macular thickness; FPR, far-peripheral region MPR, mid-peripheral region; MR, macular region; NPR, near-peripheral region; SFCT, subfoveal choroidal thickness.

Likewise, the SFCT was significantly correlated with the total CVD (R = 0.448, p < 0.001), as well as CVD in all regions except the MR (R = 0.229, *p* = 0.074 for MR; R = 0.449, *p* < 0.001 for NPR; R = 0.477, *p* < 0.001 for MPR; R = 0.305, p = 0.016 for FPR). Similarly, the thickness of Haller’s layer showed significant correlation with the total CVD (R = 0.504, *p* < 0.001) and CVD from all regions (R = 0.281, *p* = 0.027 for MR; R = 0.510, *p* < 0.001 for NPR; R = 0.520, *p* < 0.001 for MPR; R = 0.342, *p* = 0.007 for FPR). However, the thickness of the choriocapillaris-Sattler’s layer showed marginal correlation with the total CVD (R = -0.249, p = 0.051) and even in the analysis by regions also showed a significant correlation only in the NPR (R =− 0.249, *p* = 0.051 for MR; R = − 0.279, *p* = 0.028 for NPR; R =− 0.186, *p* = 0.147 for MPR; R =− 0.165, *p* = 0.201 for FPR) (Table 2).

Choroidal vascular hyperpermeability was also significantly correlated with total CVD (R = 0.481, *p* = 0.005), and CVD in all regions except the MR (R = 0.304, p = 0.091 for MR; R = 0.393, *p* = 0.026 for NPR; R = 0.473, *p* = 0.006 for MPR; R = 0.461, *p* = 0.008 for FPR).

### Comparison of clinical features according to treatment response

Based on the response to three consecutive anti-VEGF injections, 21 eyes were considered as good responders, and 11 eyes were considered as poor responders. There was no statistically significant difference between the two groups in age, sex, baseline BCVA, baseline CMT, and types of anti-VEGF applied to the patients (Table 3).

**Table 3:** Relationship between response to anti-VEGF injections and clinical characteristics in the eyes with P.

	Good responders (n = 22)	Poor responders (n = 10)	*P* value	Univariate		Multivariate	
				*B*	*P*^c^	*B*	*P*^c^
Age, years	70.2 ± 10.0	64.6 ± 8.4	0.140^a^	0.937	0.138		
Sex, n (%)			0.355^b^	0.438	0.362		
Male	14 (63.6%)	8 (80.0%)					
Female	8 (36.4%)	2 (20.0%)					
Anti-VEGF agents, n (%)			0.056^b^	1.419	0.386		
Bevacizumab	7 (31.8%)	1 (10.0%)					
Ranibizumab	7 (31.8%)	4 (40.0%)					
Aflibercept	8 (36.4%)	5 (50.0%)					
Baseline BCVA, logMAR	0.55 ± 0.35	0.40 ± 0.30	0.269^a^	0.238	0.235		
Baseline CMT, µm	415.40 ± 140.11	423.67 ± 112.31	0.764^a^	1.001	0.872		
Choroidal hyperpermeability, n (%)	5 (22.7%)	6 (60.0%)	0.040^b^	5.100	0.047	1.688	0.546
SFCT, µm	291.77 ± 81.90	371.60 ± 101.17	0.016^a^	1.010	0.036	1.014	0.022
Haller’s layer	219.59 ± 81.22	292.90 ± 97.48	0.025^a^	1.010	0.047		
Choriocapillaris-Sattler's layer	72.18 ± 24.76	78.70 ± 17.58	0.509^a^	1.014	0.448		
Choroidal vascular density, %	26.58 ± 1.77	28.39 ± 1.45	0.008^a^	1.917	0.020	2.232	0.007
Macular region (< 3 mm)	25.44 ± 2.03	26.58 ± 2.22	0.251^a^	1.312	0.169		
Near-peripheral region (3 ~ 10 mm)	25.16 ± 1.90	26.96 ± 1.61	0.009^a^	1.779	0.029		
Mid-peripheral region (10 ~ 15 mm)	28.01 ± 2.38	30.11 ± 1.81	0.018^a^	1.622	0.030		
Far-peripheral region (> 15 mm)	28.67 ± 1.80	30.99 ± 2.44	0.005^a^	1.834	0.017		

BCVA, best-corrected visual acuity; CMT, central macular thickness; PCV, polypoidal choroidal vasculopathy; SFCT, subfoveal choroidal thickness; VEGF, vascular endothelial growth factor.

^a^Mann-Whitney test, ^b^Pearson’s chi square test, ^c^Logistic regression.

**Figure 1:**
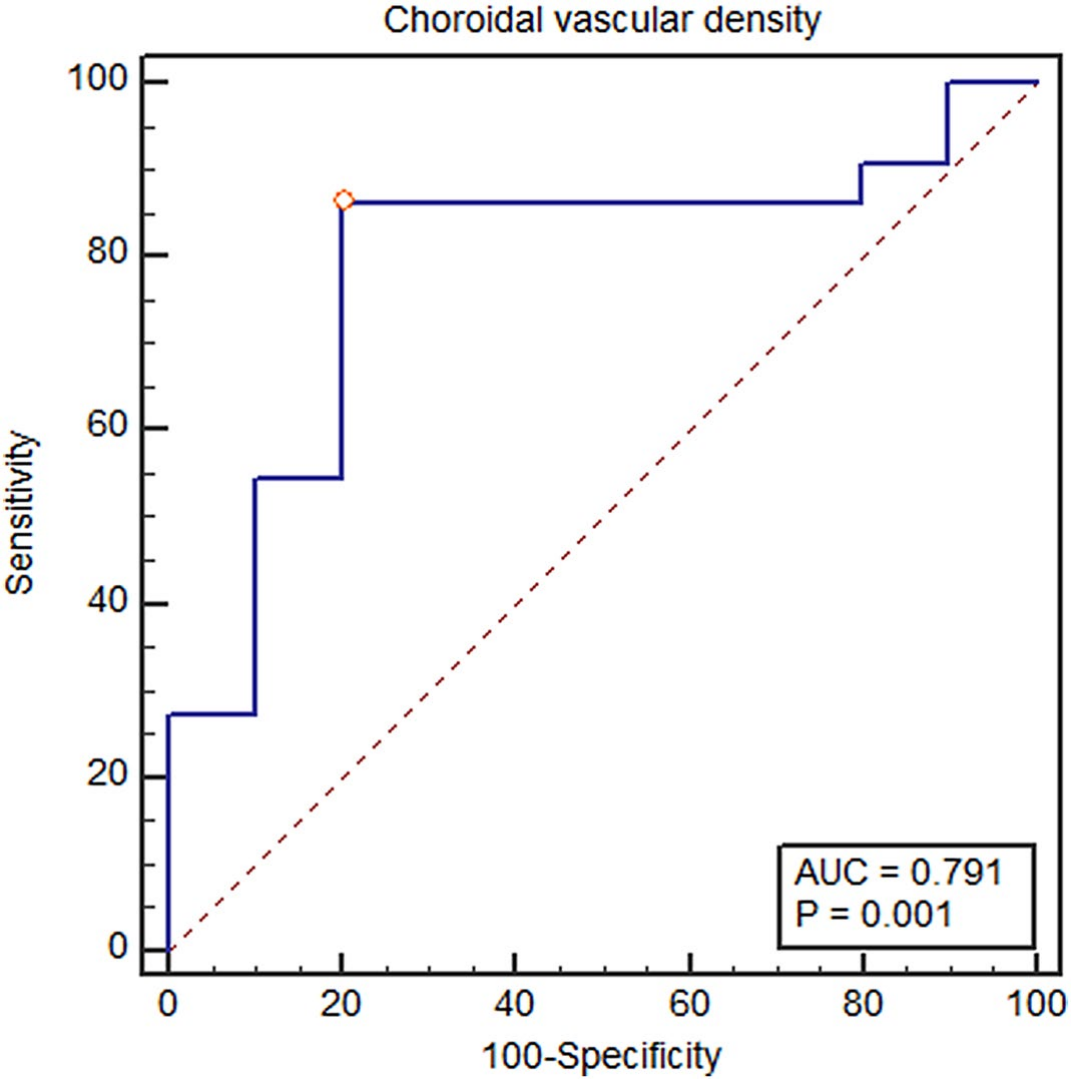
The receiver operating characteristic (ROC) curve of choroidal vascular density (CVD) and treatment response. The circled point is the cut-off point with the highest Youden index [Youden index J = 0.6636, associated criterion (cut-off) = 27.80, sensitivity = 86.4%, specificity = 80.0%]. The area under the curve was 0.791 (*p* = 0.001).

The choroid, especially in Haller’s layer, was thicker in the poor responders than in good responders (371.60 ± 101.17 µm vs. 291.77 ± 81.90 µm, *p* < 0.016 for SFCT; 292.90 ± 97.48 µm vs. 219.59 ± 81.22 µm, *p* = 0.025 for the thickness of the Haller’s layer), while the thickness of the choriocapillaris-Sattler's layer showed no statistical significant difference between the two groups (78.70 ± 17.58 µm vs. 72.18 ± 24.76 µm, *p* = 0.509) (Table 3). The choroidal vascular hyperpermeability was more frequent in the poor responders than in good responders (60.0% vs. 22.7%, *p* = 0.040) (Table 3). CVD was also significantly greater in the poor responders than good responders (28.39 ± 1.45% vs. 26.58 ± 1.77%, *p* = 0.008). CVD in all prespecified regions, except the MR, showed significant differences between the groups (26.58 ± 2.22% vs. 25.44 ± 2.03%, *p* = 0.251 for MR; 26.96 ± 1.61% vs. 25.16 ± 1.90%, *p* = 0.009 for NPR; 30.11 ± 1.81% vs. 28.01 ± 2.38%, *p* = 0.018 for MPR; 30.99 ± 2.44% vs. 28.67 ± 1.80%, *p* = 0.005 for FPR) (Table 3). Depending on the results of univariate analysis, choroidal hyperpermeability, SFCT, and CVD were subjected to binary logistic regression analysis and found that the SFCT (*p* = 0.022) and the CVD (*p* = 0.007) were independent risk factors for poor response to anti-VEGF therapy (Table 3). Based on the Youden index, the optimal cut-off point of CVD for predicting a poor treatment response was 27.80%, with a sensitivity of 86.36% and specificity of 80.00% (Fig. 1).

## Discussion

In the present study, we performed a precise measurement of CVD using stereographically projected UWF ICGA images in eyes with and without PCV. As expected, in the PCV group, the CVD increased significantly in a majority of regions, including the periphery. The CVD was positively correlated with the presence of choroidal vascular hyperpermeability and SFCT, especially in Haller’s layer. The eyes with poor treatment response after three monthly consecutive anti-VEGF injections presented relatively higher CVD than those with good response, and multivariate logistic regression analysis also showed that CVD was related to the response to anti-VEGF for PCV.

Several previous studies have revealed choroidal characteristics of PCV, including choroidal thickening, choroidal hyperpermeability, and increased choroidal vessel engorgement^5,6,10–14,22,25–28^. Consistent with the previous studies, we also found the thicker choroid and higher CVD of the PCV eyes, which showed a significant positive correlation with each other. Since ICGA can depict medium- and large-sized choroidal vessels including Haller’s layer and Sattler’s layer^29^, it is not surprising that the thickness of choroid was correlated with CVD, especially in the Haller’s layer.

In regional analysis, CVD in the peripheral area in the PCV group was significantly higher than the normal group, as in the macular area, suggesting diffuse outflow congestion of choroidal vessels in the PCV. Additionally, the increased CVD in the peripheral area was also correlated with choroidal thickness, measured at just below the fovea. These findings are in line with previous report that the increase in choroidal vasculature and hyperpermeability of PCV eyes were associated with increased ocular perfusion pressure and engorgement of the vortex vein^22,30^. Only CVD in MR, which was expected to be the most relevant, was not found to have a significant correlation with choroidal thickness. It is speculated that CVD in MR may be underestimated due to the relatively low resolution of the macula in the UWF imaging and blocked fluorescence caused by turbid subretinal fluid (SRF), subretinal hemorrhage (SRH), and pigment epithelial detachment.

However, regarding the pathogenesis of PCV, it is still uncertain whether the choroid vascular engorgement is simply an associated finding or the primary etiology of PCV. We observed that the CVD of the fellow eyes were significantly higher than the control eyes but did not differ significantly from PCV eyes. And the SFCT of the fellow eyes were significantly thicker than control eyes and significantly thinner than PCV eyes. Although the precise pathogenic mechanism regarding the choroidal changes in the PCV eye remains to be validated, the results support that diffuse choroidal venous congestion might be more suggestive of predisposing systemic background pathophysiology for the onset of PCV and the diffuse choroidal vascular dilatation may precede focal choroidal changes that cause PCV. Further longitudinal UWF ICGA studies with fellow eyes of unilateral PCV may be helpful in clarifying the pathogenesis mechanisms of PCV.

In addition, we showed the relative worse responsiveness to anti-VEGF in eyes with thicker choroid, choroidal vascular hyperpermeability, and higher CVD. CVD and SFCT were found to be the factors significantly associated with the response to anti-VEGF injections. The results are consistent with those of previous studies, which demonstrated that PCV eyes with thick choroids and choroidal hyperpermeability on ICGA show poor response to anti-VEGF treatment^9,17–21,28,31^. Baek et al. reported that baseline VEGF concentration showed a negative correlation with choroidal thickness and a positive correlation with CMT reduction after anti-VEGF treatment for PCV^32^. In the study, the authors speculated that in eyes with thick choroid, mechanical insult to the RPE may be the main factor. At the same time, an ischemic process mediated by the overexpression of VEGF may be the main factor in the development of PCV with the thin choroid. For eyes affected by PCV with higher CVD, photodynamic therapy, which induces remodeling of choroidal vessels, can be considered as an adjuvant treatment option, along with anti-VEGF injections. Further studies regarding correlations between CVD and VEGF levels in PCV are needed.

There are several limitations to this study. First, it could be underpowered to detect even small differences because of the limited sample size. Nevertheless, the strengths of this study is to provide the precise quantitative data on CVD in PCV eyes using UWF ICGA corrected for peripheral distortion, to compare them with normal controls and unaffected fellow eyes, and to evaluate treatment prognosis according to CVD. Second, while we did use a stereographic correction method, this was based on an emmetropic eye with an axial length of 24 mm^33^, and unfortunately, we did not evaluate axial length on all subjects in order to further correction. However, the mean refractory error for the subjects was 0.38 ± 1.44 and the difference in axial length could be neglected. Third, only high-quality images with good contrast were used, and this may have led to selection bias, but, at the same time, decreased information bias. Fourth, due to limited penetration of measuring light in SD-OCT, outermost layer of the choroid can be depicted dark especially in pachychoroid even using EDI and SFCT may have been measured thicker than the actual thickness. Further studies using swept-source OCT are needed in this regard.

In conclusion, CVD appeared to be higher in eyes with PCV than the normal controls in an extensive region, including the periphery. CVD of PCV eyes was positively correlated with choroidal hyperpermeability, subfoveal choroidal thickness, and CMT. These findings suggest that choroidal hypertension, leading to diffuse choroidal venous congestion, may precede PCV occurrence. Additionally, response to anti-VEGF treatment was worse in eyes with higher CVD. CVD > 27.80% could serve as an effective image biomarker that could predict poor treatment response after anti-VEGF treatment for PCV. Further, a larger sample size and a more extended period of longitudinal studies are needed to confirm these results.

## Methods

### Subjects

Patients with treatment-naïve PCV were consecutively enrolled between February 2018 and January 2019 at Yeungnam University Hospital, Daegu, South Korea. All patients voluntarily participated in the study, and written informed consent was obtained from all participants. The Institutional Review Board of Yeungnam University Medical Center (Approval Number 2017-06-011) approved the study protocols, and the research adhered to the tenets of the Declaration of Helsinki for research involving human subjects.

All subjects underwent comprehensive ophthalmic examinations, including BCVA measurement, dilated fundus examination, spectral-domain OCT (SD-OCT, Spectralis: Heidelberg Engineering, Heidelberg, Germany) with EDI, UWF fluorescein angiography (FA), and ICGA (Optos California: Optos plc, Dunfermline, United Kingdom) at baseline.

A clinical diagnosis of PCV was based on the presence of polypoidal choroidal vessels in the ICGA images. Exclusion criteria were as follows: (1) retinal disease that affects ocular circulation, such as diabetic retinopathy and retinal vein occlusion; (2) concomitant retinal disease, such as retinal detachment, macular hole, epiretinal membrane, and glaucoma; (3) refractory error more than three diopter; (4) massive SRH or fibrosis obscuring the choroidal vasculature on ICGA; (5) severe media opacity that could degrade image quality; and (6) history of previous treatments that can cause significant changes to choroidal status such as intraocular surgery or intravitreal injection. Cataract surgery performed more than three months previously was not considered an exclusion criterion.

Age-matched subjects who visited our clinic for a health-screening checkup were enrolled as normal controls without ocular disease, as confirmed by history and ophthalmic examinations. One of the two eyes in the normal control group was randomly selected.

### Choroidal vascular analysis

**Figure 2:**
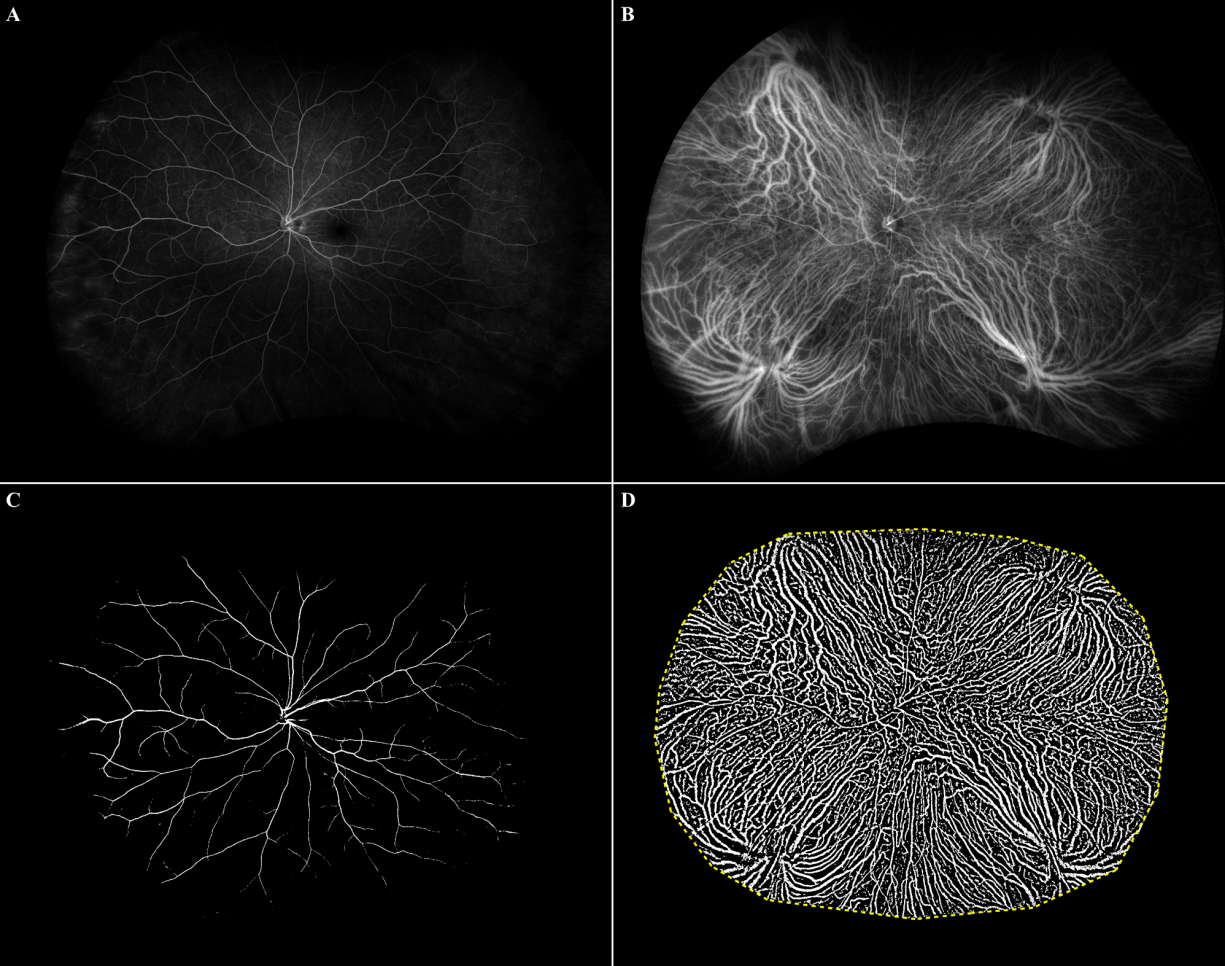
Binarization of ultra-widefield (UWF) images on fluorescein angiography (FA) and indocyanine green angiography (ICGA) in a normal eye. (**A**) UWF FA images transformed into stereographic projection images. (**B**) UWF ICGA images transformed into stereographic projection images (**C**) Binary image of UWF FA image at top-left. Retinal vascular area (RVA) from UWF FA image was automatically calculated from this image. (**D**) Binary image of UWF ICGA image. Total vascular area (TVA) from UWF ICGA image was automatically calculated from this image. Yellow dotted line represented manually outlined border of total visible area. Choroidal vascular area (CVA) was calculated by subtracting RVA from TVA.

**Figure 3:**
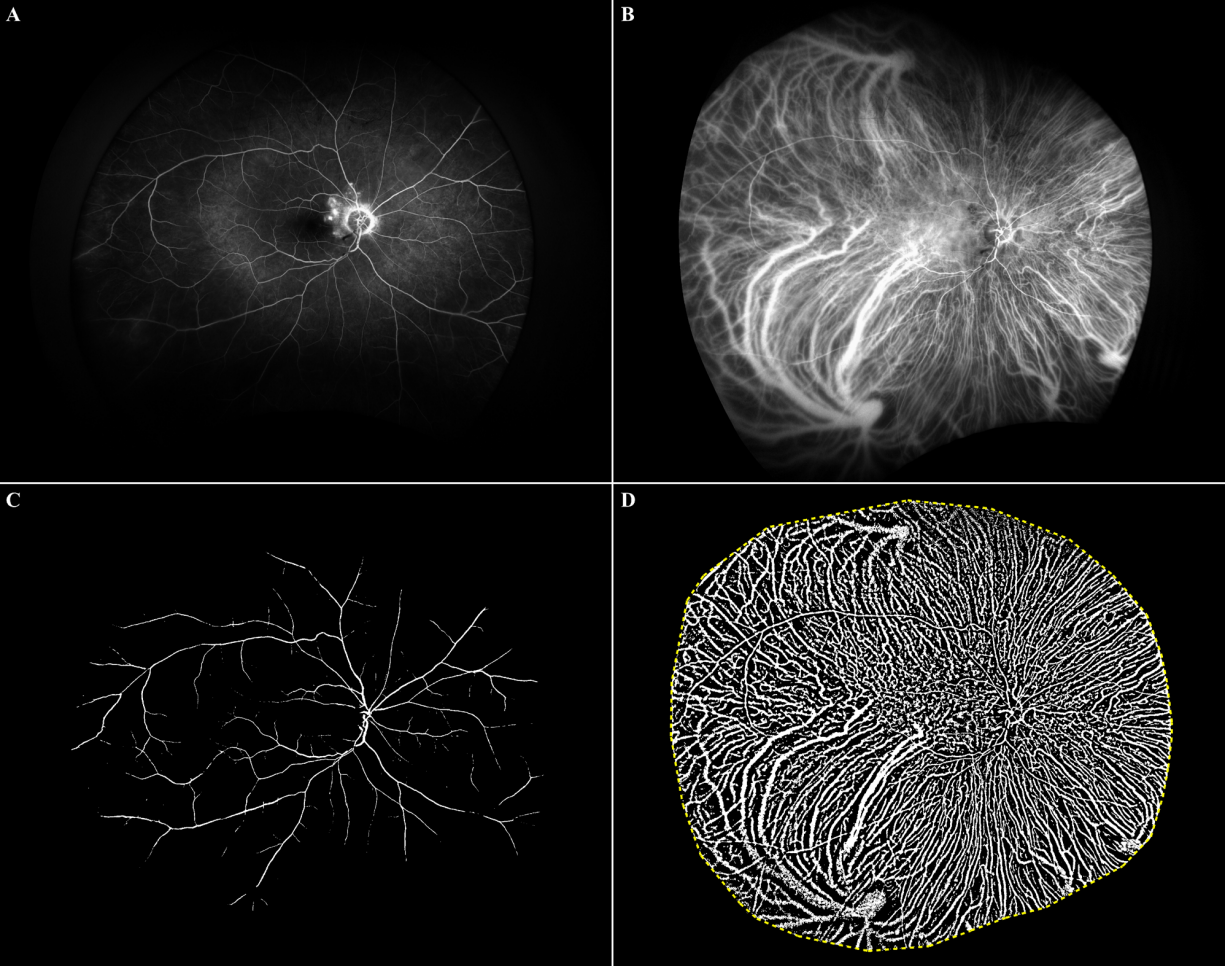
Binarization of ultra-widefield (UWF) images on fluorescein angiography (FA) and indocyanine green angiography (ICGA) in an eye with polypoidal choroidal vasculopathy. (**A**) UWF FA images transformed into stereographic projection images. (**B**) UWF ICGA images transformed into stereographic projection images (**C**) Binary image of UWF FA image at top-left. Retinal vascular area (RVA) from UWF FA image was automatically calculated from this image. (**D**) Binary image of UWF ICGA image. Total vascular area (TVA) from UWF ICGA image was automatically calculated from this image. Yellow dotted line represented manually outlined border of total visible area. Choroidal vascular area (CVA) was calculated by subtracting RVA from TVA.

UWF FA and ICGA were performed simultaneously after intravenous injection of 5 mL of 10% sodium fluorescein and 2 mL of 25 mg of indocyanine green. The images in the early phase (two minutes after dye injection) were used for the extraction of choroidal vasculature. Images were transformed into stereographic projection images using the manufacturer’s software. After processing with a top-hat filter, the FA and ICGA images were binarized^29^, with noise removed. Finally, the binarized images were trimmed and evaluated for maximum area, excluding all of the following conditions, such as areas covered by eyelashes, low contrast areas obscuring retinal vasculature in UWF FA, and choroidal vasculature in UWF ICGA. Total vascular area (TVA) from the ICGA image and retinal vascular area (RVA) from the FA image were calculated in mm^2^ by each pixel from the binarized vascular area^34^. CVA was calculated by subtracting RVA from TVA (Figs. 2 and 3). CVD was also calculated by dividing CVA by total visible area, in which the peripheral extent of the blood vessel arborization was manually outlined. Two masked retinal specialists (C.M. and G.R.) analyzed the images and calculated CVA and CVD using ImageJ version 1.51j8 (National Institute of Health, Bethesda, MD, USA). The average values from the two independent gradings were used. Regional distributions of CVA and CVD were assessed using a grid with several concentric rings centered on the fovea which was applied to define four zones: MR (0.5–3 mm radius), NPR (3–10 mm), MPR (10–15 mm), and FPR (15 mm-outer gradable perfusion boundary).

Images in the mid- and late-phase (> 5 min after dye injection) were used to evaluate the incidence of choroidal vascular hyperpermeability. Choroidal vascular hyperpermeability was determined in eyes with PCV if there were multifocal hyperfluorescent areas with indistinct margins from choroidal vasculature in the mid- to late-phase ICGA.

### Subfoveal choroidal thickness measurement

**Figure 4:**
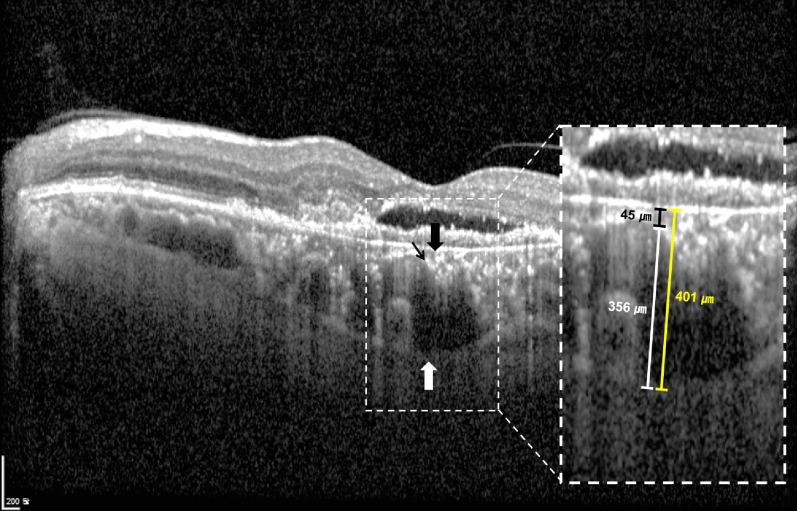
Measurement of subfoveal choroidal thickness (SFCT) and the thickness of choroidal substructures with an enhanced depth imaging optical coherence tomography. SFCT was measured vertically from the hyper-reflective line of Bruch’s membrane (black thick arrow) to the line connecting the outer margin of the large choroidal vessel layer (white thick arrow). The thickness of Haller’s layer was measured vertically from the line connecting the inner margin of the large choroidal vessel layer (black thin arrow) to the line connecting the outer margin of that (white thick arrow). The thickness of choriocapillaris-Sattler’s layer was calculated by subtracting the thickness of Haller’s layer from the total choroidal thickness.

The choroidal thickness was measured using EDI-OCT. The thickness values of the total choroid, choriocapillaris-Sattler’s layer, and Haller’s layer were measured in the subfoveal region (Fig. 4). The SFCT was defined as the vertical distance from the hyper-reflective line of Bruch’s membrane to the line connecting the outer margin of the large choroidal vessel layer. The thickness of Haller’s layer was defined as the vertical distance from the line connecting the inner margin of the large choroidal vessel layer to the line connecting the outer margin. The thickness of the choriocapillaris-Sattler’s layer was calculated by subtracting the thickness of Haller’s layer from the total choroidal thickness. Measurements were performed using the built-in caliper tool (Heidelberg Eye Explorer, Version 1.9.10, Heidelberg Engineering) at a single point below the fovea by two independent masked graders (C.M. and G.R.). When there was a disagreement between the graders, the supervising grader (M.S.) confirmed the final decision.

### Treatment response

For patients with PCV, intravitreal anti-VEGF injections were performed using bevacizumab (Avastin: Roche, Kaiseraugst, Switzerland), ranibizumab (Lucentis: Genentech, San Francisco, CA), or aflibercept (Eylea: Regeneron, Tarrytown, NY). The selection of anti-VEGF agents was remained at the discretion of the treating physician. All patients were treated following a protocol that included a loading dose with three consecutive intravitreal injections of the same anti-VEGF agent at 1-month intervals. The good responders were defined as those who showed complete resolution of SRF and/or intraretinal fluid (IRF) on OCT at a month after three consecutive anti-VEGF injections. The poor responders were defined as those who showed remained SRF and/or IRF on OCT after the three consecutive injections.

### Statistical analysis

The sample size was estimated by the free-software G power (version 3.1.9.4, Franz Faul, University of Kiel, Kiel, Germany). With a power of 80%, a significance level of 0.05, and effect size of 0.8, the sample size for each group was calculated to be 26. Considering dropouts, we planned to include more than additional 10% of calculated sample size.

Statistical analyses were performed using IBM SPSS V.20.0 for Windows (IBM Co., Armonk, New York, USA). The Mann–Whitney U test and the Chi-square test were used to compare numerical variables between PCV patients and age-matched healthy controls and numerical variables between good responders and poor responders, respectively. The Pearson correlation test was performed between numerical variables and CVD from each region. The Youden index (defined as “sensitivity + specificity -100”) was used to determine the optimal CVD cut-off point for the best discrimination between the good and poor responders. Variables with a p-value of less than 0.05 were considered statistically significant. All results were presented as the mean ± standard deviation.

## Data Availability

The datasets generated during and/or analysed during the current study are available from the corresponding author on reasonable request.
